# Glycodelin A is a prognostic marker to predict poor outcome in advanced stage ovarian cancer patients

**DOI:** 10.1186/1756-0500-5-551

**Published:** 2012-10-05

**Authors:** Christoph Scholz, Sabine Heublein, Miriam Lenhard, Klaus Friese, Doris Mayr, Udo Jeschke

**Affiliations:** 1 Department of Obstetrics and Gynecology, Heinrich-Heine University, Düsseldorf, Germany; 2 Department of Obstetrics and Gynecology, Campus Innenstadt, Ludwig-Maximilians-University Munich, Munich, 80337, Germany; 3 Department of Obstetrics and Gynecology, Campus Großhadern, Ludwig-Maximilians-University Munich, Munich, 80337, Germany; 4 Department of Pathology, Ludwig-Maximilians-University Munich, Munich, 80337, Germany

**Keywords:** Ovarian cancer, Glycodelin, Immunohistochemistry, Prognosis

## Abstract

**Background:**

Glycodelin is a cell surface glycoprotein offering a unique gender specific carbohydrate configuration. Sialylated carbohydrate structures, which are unusual for mammals, characterize Glycodelin isolated from amniotic fluid (Glycodelin A, GdA). Glycodelin in general exerts multiple, partly opposing functions ranging from immunosuppression to cell differentiation. As these markedly influence tumorigenesis, this study aimed to clarify whether expression of different Glycodelin isoforms is related to clinicopathological characteristics and prognosis of ovarian cancer patients. Further the use of Glycodelin as a serum marker in benign and malignant ovarian diseases was evaluated.

**Methods:**

Ovarian cancer specimens (n = 152) were stained for Glycodelin with carbohydrate and peptide specific antibodies. Associations between Glycodelin expression and histological grading, FIGO stage as well as patient’s prognosis were examined. Glycodelin was correlated to expression of gonadotropin receptors and mucin-1, which are discussed as ovarian cancer tissue markers. In addition, Glycodelin serum concentrations were analyzed in patients suffering from benign (n = 73) or malignant (n = 38) ovarian neoplasias.

**Results:**

Glycodelin A was found to be an independent prognostic marker for poor prognosis in advanced ovarian cancer patients. GdA staining correlated with gonadotropin receptor (FSHR and LHCGR) and with hCG expression. Gd expression showed a positive correlation with a tumour-associated epitope of mucin 1 (TA-MUC1). Further, compared to ovarian cancer, serum Gd was increased in patients with benign ovarian tumors.

**Conclusion:**

Glycodelin A might be related to tumor aggressiveness and poor clinical outcome in advanced epithelial ovarian cancer. Glycodelin serum levels found in patients suffering from benign ovarian tumors, might contribute to a more global attenuation during progression of these precursor lesions.

## Background

Epithelial ovarian cancer (EOC) represents the most lethal malignancy of the female genital tract. Nowadays ovarian cancer patients’ prognosis mostly relies on completeness of surgical tumor resection [1,2], clinical staging and histological tumor grading[3]. Thus there is a compelling need to identify and validate tumor specific antigens which are suitable to individualize therapeutic strategies. Interestingly, during EOC evolvement and progression host anti-tumor immune defense seems to be actively blocked by tumor derived mediators. By creating this highly suppressive environment, EOC manages to extensively grow and spread in the peritoneal cavity.

Glycodelin (Gd), a potent immunosuppressive agent of the reproductive tract, is supposed to contribute to this immune tolerant phenotype. Gd is a glycoprotein whose immune-regulatory actions have been highlighted within different biological processes [4-6] and which is abundantly found in the female reproductive tract [7-9]. Structure wise it is part of the lipocalin superfamily and exerts its potent immune-regulatory activity via its unique, heavily sialysiated glycosylation pattern. Apart from its physiologic role as an immunomodulatory agent during implantation of the fetal semiallotransplantant it is also expressed by malignant tissues and contributes to the tumor-micromilieu [10,11]. Nevertheless, the physiological importance of Gd-expression in malignant diseases remains unknown. Gd is one of very few proteins that show a gender specific glycosylation pattern. Glycodelin, isolated from amniotic fluid (glycodelin A, GdA) is composed of two identical subunits closely connected by non-covalent bonds and a carbohydrate content of 17.5% [12]. A similar glycoprotein, Glycodelin S (GdS), was found in seminal plasma, but with a different glycosylation compared to GdA. While GdA is heavily sialylated, GdS is characterized by fucose-rich carbohydrate structures [13].

In the current study Gd was detected by antibodies raised against peptide sequences, which are not gender specific or specific for GdA or GdS, and a GdA specific monoclonal antibody [14,15]. In this work we aimed to clarify whether Gd expression in EOC is of prognostic significance. Further Glycodelin was correlated to expression of gonadotropin receptors and Mucin-1, which are discussed as ovarian cancer tissue markers. Finally we tested whether Glycodelin might be a potentially useful serum biomarker to detect ovarian cancer.

## Materials and methods

### Tissue acquisition

All tissue samples (n = 152) were got at surgery for primary EOC in patients treated at the Department of Obstetrics and Gynecology of the Ludwig-Maximilians-University Munich between 1990 and 2002. Specimens were assessed by two gynecological pathologists according to the criteria of the FIGO and the World Health Organization (WHO). Follow up data, which were received from the Munich Cancer Registry, and patients’ characteristics are listed in Table 1.

**Table 1:** Patients’ characteristics; Details on patients included in immunohistochemistry (A) and EIA study (B) are shown

**Stage**	**Patients (n)**
**I**	34
**II**	10
**III**	100
**IV**	3
**n.a.**	5
**Grade**	
**G1**	37
**G2**	50
**G3**	53
**n.a.**	12
**deaths**	102
	
**cystic lesions**	21
**inflammatory diseases**	18
**benign tumors**	34
**malignant tumors**	38
**total**	111

n.a. = data on grade/FIGO stage were not available.

Sera of 111 patients, who underwent surgery at the Department of Obstetrics and Gynecology of Ludwig-Maximilians-University Munich between 2002 and 2005, were collected before surgery. Histological diagnoses (Table 1 B, benign ovarian diseases, n = 73 and EOC, n = 38) were made by gynecological pathologists. Written informed consent was obtained from all patients before surgery. Benign ovarian diseases were set up of cystic lesions (n = 21; serous cysts, mucinous cysts, follicle cysts, inclusion cyst, corpus luteum cysts), inflammatory diseases (n = 18; endometriosis cysts, sactosalpinx) and benign tumors (n = 34; serous and mucinous cystadenofibroma, fibroma, teratoma). Sera of EOC patients (n = 38) were analyzed in parallel.

### Immunohistochemistry

Tissue specimens were dewaxed in xylol and endogenous peroxidase was quenched with 3% hydrogen peroxide for 20 min. Following epitope retrieval, slides were blocked and antibody staining was performed using the Vectastain elite kit (Vector Laboratories, Burlingame, USA) according to the manufacturer’s protocol. For details on blocking and staining procedure see Table 2. Finally, slides were stained with diaminobenzidine (Dako, Hamburg, Germany), counterstained in Mayer's acidic hematoxylin and cover slipped with Consul Mount (Thermo Shandon, Pittsburgh, PA). Isotype matched mouse IgG instead of the primary antibody was used as a negative control and tissue positive controls are listed in Table 2. The signal was quantified using a semi quantitative method [16] by two examiners. At a glance the immuno-reactive (IR)-score quantifies staining intensity (1 = low, 2 = moderate, 3 = strong) and percentage of stained cells (0 = no, 1 = less than 10%, 2 = 10% - 50%, 3 = 51% - 80%, 4 = 81% - 100% stained cells). Multiplication of these values results in the IR-score ranging from 0 (negative) to 12 (strongly positive). Antibody staining used to calculate Spearman’s rho are either published [17,18] (LHCGR, FSHR, hCG) or are in press (PankoMabGEX™).

**Table 2:** Antibodies used for immunohistochemistry and EIA; GdC15, GdQ13 and GdN20 were purchased from SantaCruz Biotechnologies, Santa Cruz, CA (SCBT)

**Immunohistochemistry**							
**Antibody**	**Epitope**	**Source/ Clonality**	**Epitope retrieval**	**Blocking**	**Dilution**	**Incubation**	**Reaction system used for detection**
GdC15 (SCBT)	C-terminal	goat/polyclonal	Proteinase K (30 min, RT)	UV-Block (45 min)	1:1000 (UV Block)	30 min (RT)	Vectastain elite kit (goat IgG)
GdQ13 (SCBT)	C-terminal	goat/polyclonal	citrate buffer (pH 6, 5 min, pressure cooker)	UV-Block (45 min)	1: 300 (UV Block)	o.n. (4°C)	Vectastain elite kit (goat IgG)
GdA (Jeschke *et al.* 2006)	Mixed glycan/protein epitope	mouse/ monoclonal	citrate buffer (pH 6, 5 min, pressure cooker)	1.5% horse serum (20 min)	1:3000 (DAKO dil.)	o.n. (4°C)	Vectastain elite kit (mouse IgG)
**Competitive enzyme immuno assay (EIA)**							
**Antibody**	**Epitope**	**Source/ Clonality**	**Competing peptide**	**Blocking**	**Dilution**	**Incubation**	**Reaction system used for detection**
GdQ13 (SCBT)	C-terminal	goat/ polyclonal	GdQ13-P (SCBT)	1% BSA, 0.5% NaN_3_ in PBS	1.5 ng/ml	1 hour (RT)	POD-strepavidin
GdN20 (SCBT)	N-terminal	goat/ polyclonal	GdN20-P (SCBT)	1% BSA, 0.5% NaN_3_ in PBS	6 ng/ml	1 hour (RT)	POD-strepavidin

rt = room temperature, BSA = bovine serum albumin, PBS = phosphate buffered salt solution, o.n. = over night, POD = horse radish peroxidase.

Competing peptides came from SCBT as well and were biotinylated by the manufacturer; unmodiefied GdQ13-P, GdN20-P were used for setting up the standard curve. Preparation of GdA is published in Jeschke *et al.* 2006. Epitope retrieval: Proteinase K was from Quiagen (Hilden, Germany), citrate buffer contained 0.1 M citric acid and 0.1 M sodium citrate in distilled water (pH 6.0). Blocking: UV-Block was purchased from Thermo Scientific (Bonn, Germany) and horse serum was a component of the Vectastain elite kit (Vector Laboratories, Burlingame, USA).

### Analysis of Gd serum levels

Sera were collected prior to surgery and stored at - 80°C. Analysis was performed using a competitive enzyme immune assay (EIA) principle. Briefly, plates were coated with antibodies (Table 2), which were rose against a peptide sequence localized in the C-terminal (GdQ13) or N-terminal (GdN20) end of the Gd protein core, over night at RT and blocked in blocking solution for 30 min. Following washes with PBS plates were incubated with biotinylated Gd standard peptides (GdN20 peptid 6 ng/ml; GdQ13 peptid 1.5 ng/ml) for 1 hour followed by washes in PBS. Serum was diluted 1/2 in PBS and applied onto the plate for 1 hour to perform the competition step. After washes with PBS the plate was incubated with POD streptavidine for 2 min before the reaction was stopped with 1 N sulphuric acid. The readout was performed using an ELISA Reader Dynex MRX (Dynex Technologies, Chantilly, VA). Amniotic fluid or just blocking solution was used as positive and negative controls, respectively.

### Statistical analysis

Data were analysed employing the SPSS (v19, IBM, Armonk, New York) statistic software for MS windows. Statistical significance for pair wise comparisons of unlinked non parametric values was determined by Mann–Whitney-U test whereas differences among three or more groups were tested using Kruskal-Wallis one-way analysis of variance by ranks, respectively. Survival and recurrence free survival was plotted in accordance with Kaplan-Meier survival analysis. Correlations were assessed using Spearman’s rho and gamma correlation coefficient for two independent variables. Statistical significance for all tests was assumed for p < 0.05 and data are presented as mean ± standard error.

## Results

### Patients’ characteristics and Glycodelin expression

34 patients presented with early disease in stage I (FIGO I). 10 patients had FIGO stage II and 103 patients underwent surgery because of suspected ovarian cancer involving the peritoneal cavity (100 patients FIGO stage III and 3 patients stage IV). All patients who suffered from EOC staged FIGO II - IV received carboplatin and paclitaxel as chemotherapy after surgery. Histological 53 tumors were assigned with WHO grade G3, 50 with G2 and another 37 were classified as G1. Mean follow-up time was 11.03 ± 0.58 years and mean overall survival 7.12 ± 0.63 years. 27 relapses and 102 deaths were documented.

**Figure 1:**
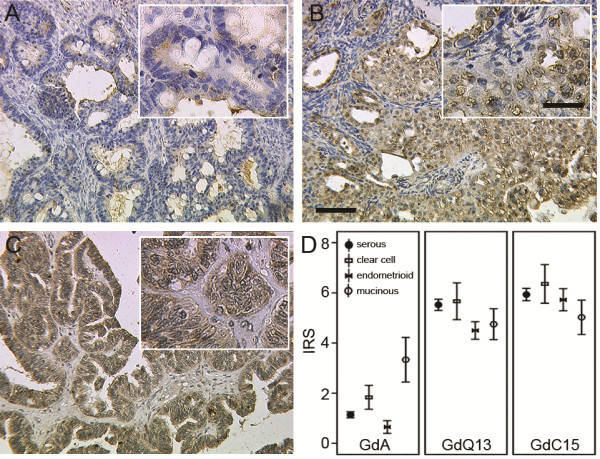
**Immunohistochemical staining of GdA (A), GdQ13 (B) and GdC15 (C).** Representative images of GdA (**A**), GdQ13 (**B**) and GdC15 (**C**) are shown. GdA expression in mucinous cancers was significantly higher than in the remaining entities (D; p = 0.007). Mean IR-scores for GdA, GdQ13 and GdC15 are shown (**D**); error bars represent standard error. Scale bar in B equals 100 μm (50 μm in insert) and applies to A - C.

Gd was detected by three different antibodies (Figure 1), which recognize distinct peptide epitopes of Gd or target GdA specifically. Both peptide antibodies showed strong to moderate staining (mean IRS GdQ13 = 5.35 ± 0.18; mean IRS GdC15 = 5.86 ± 0.20) which was equally distributed within the four EOC subtypes examined (Figure 1 B, C, D). However GdA staining in general was much less intense (mean IRS = 1.31 ± 0.13) and showed significantly different staining within subtypes (p = 0.004) and highest expression (p = 0.007) in mucinous (mean IRS = 3.33 ± 0.89) compared to serous, clear cell and endometrioid carcinomas (Figure 1 A, D). Interestingly, Gd staining (IRS > 0) determined by the peptide specific antibodies (GdC15, GdQ13) was found in 100% of the tissue sections examined, while GdA signal was just detected in 58.6%.

### Correlation of Glycodelin with tumor biomarkers, clinicopathological criteria and patients’ prognosis

For the peptide specific antisera GdC15 and GdQ13 a strong correlation in between the two (Table 3) was observed (p < 0.001). Expression of GdC15, GdQ13 and GdA epitopes rose with positivity for PankoMabGEX™ (GdC15, p = 0.007; GdQ13, p = 0.010; GdA, p = 0.004), which recognizes a special tumour-associated epitope on MUC1, TA-MUC1, that either localizes to the cell membrane (mPankoMabGEX™) or cytoplasm (cPankoMabGEX™). Furthermore GdA staining correlated with gonadotropin receptor (FSHR, p = 0.031; LHCGR, p = 0.048) and hCG (p = 0.027) expression. Statistical analysis revealed no correlation of either GdA or GdQ13 antibodies staining with known prognostic markers in EOC, namely histological grade or FIGO stage. GdC15 was positively correlated to FIGO stage (p = 0.048).

**Table 3:** Correlations between GdC15, GdQ13 and GdA; Correlations between GdC15, GdQ13 and GdA were assessed using Spearman’s rho test for two independent variables

			**GdA**	**GlycC15**	**GlycQ13**	**LHCGR**	**FSHR**	**hCG**	**mPankoMabGEX**	**cPankoMabGEX**
Spearman's rho	GdA	Correlation Coefficient	1.000	-.142	-.054	.162^*^	.178^*^	.187^*^	.045	.241^**^
		Sig. (2-tailed)	—	.081	.508	.048	.031	.027	.596	.004
	GlycC15	Correlation Coefficient	-.142	1.000	.456^**^	-.027	.155	-.045	.228^**^	-.094
		Sig. (2-tailed)	.081		< .001	.740	.061	.598	.007	.270
	GlycQ13	Correlation Coefficient	-.054	.456^**^	1.000	.118	.080	.047	.217^**^	-.128
		Sig. (2-tailed)	.508	< .001	—	.152	.338	.585	.010	.131

A correlation coefficient is presented and statistical significance for all tests was assumed for p < 0.05. * represents p < 0.05, ** represents p < 0.01.

### Glycodelin A predicts prognosis in EOC patients

**Figure 2:**
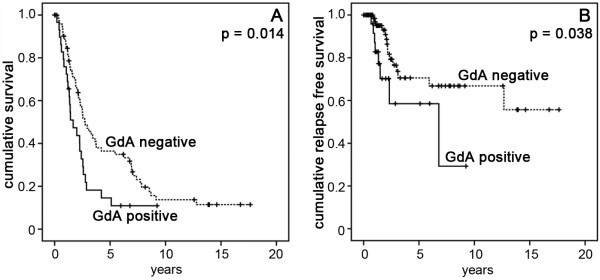
**Cumulative survival (A), and cumulative recurrence free survival (B) of patients who underwent surgery for EOC, were plotted in accordance with Kaplan-Meier survival analysis.** Patients who presented with advanced stage (FIGO III, IV) cancer had significantly less favorable prognosis if their tumor expressed GdA (**A**) and their cumulative recurrence free survival was also significantly shortened (**B**). Statistical significance for all tests was assumed for p < 0.05.

Whether Gd protein expression is useful to predict patients’ prognosis was analyzed by drawing Kaplan-Meier plots. Low expression - in respect of median expression IR-scores - was defined as IRS (GdA) ≤ 1; IRS (GdQ13) ≤ 4; IRS (GdC15) ≤ 4. In Kaplan-Meier analysis patients with GdA-positive advanced EOC (stage III and IV) had a significantly poorer overall-survival (log rank, p = 0.014, Fig. 2 A) and five-year survival (log rank, p = 0.009) than patients with GdA-negative tumors. In these patients with advanced stage ovarian cancer GdA positivity predicted significantly shorter recurrence free survival (log rank, p = 0.038) when compared to GdA negative tumors (Figure 2 B). In stage I and II no such difference could be shown. Further, GdA expression was analyzed in advanced staged patients whose survival after first diagnosis was longer than ten years (n = 7). Interestingly, none of these cases was found to be positive for GdA. Neither GdC15 nor GdQ13 were related to patients’ prognosis regarding overall survival or recurrence free survival, respectively.

### Serum Glycodelin concentrations

**Figure 3:**
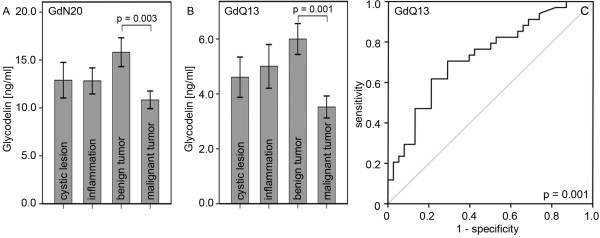
**Analysis of Gd serum concentrations by EIA.** Gd concentrations were determined by two different EIA assays using GdN20 (**A**) and GdQ13 (**B**). Bar charts represent mean Gd concentrations [ng/ml] and error bars represent standard errors. Gd concentrations in sera derived from patients with benign and malignant ovarian tumors as measured by GdQ13-EIA are plotted as ROC-curve (**C**). Statistical significance for all tests was assumed for p < 0.05.

Sera (n = 111) came from patients either presenting with benign (n = 73) or malignant (n = 38) ovarian diseases. Ovarian cancer patients mostly presented at stage III (FIGO I: 16.0%, FIGO II: 12.0%, FIGO III: 56.0% and FIGO IV: 16.0%). Benign neoplasias were further sub classified into simple ovarian cysts (n = 21), endometriosis and inflammatory diseases (n = 18) and benign ovarian tumors (n = 34). Serum Gd concentrations were determined by EIA using the two peptide antibodies GdN20 and GdQ13 (Figure 3 A, B) for targeting the N - and C-term of Gd, respectively. Gd was detected in 100% (GdN20) and 99.1% (GdQ13) of all sera. Significantly higher Gd concentrations (Figure 3 A, B) were found in sera collected from patients with benign ovarian tumors when compared to those with ovarian cancer (GdN20: p = 0.003, 10.84 ± 0.92 ng/ml vs. 15.81 ± 1.50 ng/ml and GdQ13: p = 0.001, 3.53 ± 0.40 ng/ml vs. 6.00 ± 0.56 ng/ml). GdQ13 (Figure 3 C) and GdN20 turned out to sensitively distinguish sera of patients with benign ovarian tumors with acceptable specificity (Figure 3 C; GdQ13: AUC = 0.729 ± 0.59, p = 0.001, 95% CI: 0.61 - 0.85; GdN20: AUC = 0.705 ± 0.06, p = 0.003, 95% CI: 0.58 - 0.83). No significant differences in Gd serum concentration were observed between patients presenting with malignant ovarian cancer, inflammatory ovarian diseases or ovarian cysts.

## Discussion

This study found that GdA independently predicts unfavorable prognosis in advanced stage ovarian cancer patients. Gd has already been published to correlate with less favorable clinical presentation in breast [19] and lung cancer [20], though controversial results were observed in ovarian cancer patients [21]. However a common problem in research on Gd is that most commercially available antisera were raised against peptide epitopes. Since gender specific glycosylation pattern crucially determines Gd-protein function [22], we employed GdA specific staining [14,15]. Remarkably, just GdA correlated with patients’ prognosis (Figure 2) and GdA was much more selectively distributed than the peptide epitopes (Figure 1). Thus we hypothesize, that the specific glycosylation pattern of GdA and not necessarily just its presence as determined by the peptide specific antisera, is responsible for its tumor promoting activity. Therefore GdA might be an interesting marker to discriminate between high risk and low risk patients in advanced ovarian cancer (stage III and IV). A former study showed that Gd is also secreted to ascites fluid of ovarian cancer patients and that this Gd has significant differences in its structure of sialyl Lewis-type oligosaccharides compared to GdA. Additionally, ascites Gd inhibits IL-2 stimulated proliferation of peripheral blood leucocytes and inhibits adhesion of SLeX-positive cells to E-selectin. Therefore, Gd could act as an inhibitor of lymphocyte activation and/or adhesion in ovarian cancer [23].

As several studies proofed the immune suppressive effect of GdA [6,11], we suppose that GdA interferes with host anti tumor immune defense in EOC as well. Besides, it is widely accepted, that EOC has the ability to escape immune rejection by creating an immune suppressive environment [24]. Thus expression of the immunomodulatory GdA in ovarian carcinomas might contribute to permit immunological destruction of malignant cells and therefore might be related to tumor aggressiveness and poor clinical outcome.

Furthermore GdA staining correlated with gonadotropin receptor (FSHR and LHCGR) and with hCG expression itself (Table 3). There are only few studies on human chorionic gonadotropin and its receptor expression in ovarian cancer tissue [25,26]. In a recent study we found an opposed prognostic value of LHCGR and FSHR in ovarian cancer [17] in almost the same panel of patients as described within this study. GdA and hCG stimulate each other in a positive feedback mechanism in the placental decidua/trophoblast cell system as described earlier [27,28]. Results obtained within this study seem to confirm former results of GdA - hCG interaction. Gd expression showed also a positive correlation with a tumour-associated epitope of MUC1 (TA-MUC1), which was stained by the PankoMabGEX™ antibody. This epitope was found to be steroid hormone receptor dependent in breast cancer [29]. Because it is known that Gd expression is also regulated by steroid hormones [30], there could be a common regulation pathway for both Gd and TA-MUC1.

The use of Gd as a serum biomarker in ovarian cancer patients has been controversially discussed [31,32]. As Gd might interfere with tumor development, we decided to examine precursor lesions as well, which turned out to be characterized by elevated serum Gd levels. Interestingly in sera collected from ovarian cancer patients Gd levels were not significantly altered compared to cystic or inflammatory diseases. Benign ovarian tumors are part of a multistep process leading to low grade invasive ovarian cancer [33]. Gd mediated immune attenuation has been described during pregnancy [34-36] as well as in inflammatory processes [37]. Thus, besides of it pathophysiologic role, Gd might be an interesting to detect benign or inflammatory changes in the ovaries, but the detection of GdA with its unique glycosylation may be a more suitable serum marker for ovarian cancer patients.

## Conclusion

GdA is related to tumor aggressiveness and poor clinical outcome in advanced ovarian cancer. GdA might be an interesting marker to discriminate between high risk and low risk patients in stage III and IV ovarian cancer.

## Competing interests

There is no financial or personal interest in relation to the work described.

## Authors’ contributions

CS, SH and ML have made substantial contributions to conception, design and acquisition of data. DM has made substantial contributions to analysis and interpretation of data, and has been involved in drafting the manuscript and revising it critically for important intellectual content. KF and UJ have given final approval of the version to be published. In addition, DM and UJ have made substantial contributions to conception and design of the study. All the authors have read and approved the manuscript for publication.

## References

[B1] Classe JM Jaffre I Frenel JS Bordes V Dejode M Dravet F Ferron G Marchal F Berton Rigaud D Loussouarn D Prognostic factors for patients treated for a recurrent FIGO stage III ovarian cancer: a retrospective study of 108 cases Eur J Surg Oncol 2011 37 11 971 977 10.1016/j.ejso.2011.08.138 21944959

[B2] Bristow RE Tomacruz RS Armstrong DK Trimble EL Montz FJ Survival effect of maximal cytoreductive surgery for advanced ovarian carcinoma during the platinum era: a meta-analysis J Clin Oncol 2002 20 5 1248 1259 10.1200/JCO.20.5.1248 11870167

[B3] Lenhard SM Bufe A Kumper C Stieber P Mayr D Hertlein L Kirschenhofer A Friese K Burges A Relapse and survival in early-stage ovarian cancer Arch Gynecol Obstet 2009 280 1 71 77 10.1007/s00404-008-0877-z 19093129

[B4] Lee CL Lam KK Koistinen H Seppala M Kurpisz M Fernandez N Pang RT Yeung WS Chiu PC Glycodelin-A as a paracrine regulator in early pregnancy J Reprod Immunol 2011 90 1 29 34 10.1016/j.jri.2011.04.007 21641661

[B5] Ren S Liu S Howell PM Jr Zhang G Pannell L Samant R Shevde-Samant L Tucker JA Fodstad O Riker AI Functional characterization of the progestagen-associated endometrial protein gene in human melanoma J Cell Mol Med 2010 14 6B 1432 1442 19799645 10.1111/j.1582-4934.2009.00922.x PMC3829010

[B6] Scholz C Toth B Brunnhuber R Rampf E Weissenbacher T Santoso L Friese K Jeschke U Glycodelin A induces a tolerogenic phenotype in monocyte-derived dendritic cells in vitro Am J Reprod Immunol 2008 60 6 501 512 10.1111/j.1600-0897.2008.00647.x 19032611

[B7] Jeschke U Toth B Scholz C Friese K Makrigiannakis A Glycoprotein and carbohydrate binding protein expression in the placenta in early pregnancy loss J Reprod Immunol 2010 85 1 99 105 10.1016/j.jri.2009.10.012 20299109

[B8] Scholl B Bersinger NA Kuhn A Mueller MD Correlation between symptoms of pain and peritoneal fluid inflammatory cytokine concentrations in endometriosis Gynecol Endocrinol 2009 25 11 701 706 10.3109/09513590903159680 19903048

[B9] Bersinger NA Birkhauser MH Yared M Wunder DM Serum glycodelin pattern during the menstrual cycle in healthy young women Acta Obstet Gynecol Scand 2009 88 11 1215 1221 10.3109/00016340903294264 19900139

[B10] Koistinen H Hautala LC Seppala M Stenman UH Laakkonen P Koistinen R The role of glycodelin in cell differentiation and tumor growth Scand J Clin Lab Invest 2009 69 4 452 459 10.1080/00365510903056023 19551557

[B11] Scholz C Rampf E Toth B Brunnhuber R Weissenbacher T Friese K Jeschke U Ovarian cancer-derived glycodelin impairs in vitro dendritic cell maturation J Immunother 2009 32 5 492 497 10.1097/CJI.0b013e3181a59fa9 19609241

[B12] Bohn H Kraus W Winckler W New soluble placental tissue proteins: their isolation, characterization, localization and quantification Placenta Suppl 1982 4 67 81 6085895

[B13] Koistinen H Koistinen R Dell A Morris HR Easton RL Patankar MS Oehninger S Clark GF Seppala M Glycodelin from seminal plasma is a differentially glycosylated form of contraceptive glycodelin-A Mol Hum Reprod 1996 2 10 759 765 10.1093/molehr/2.10.759 9239694

[B14] Jeschke U Bischof A Speer R Briese V Richter DU Bergemann C Mylonas I Shabani N Friese K Karsten U Development of monoclonal and polyclonal antibodies and an ELISA for the determination of glycodelin in human serum, amniotic fluid and cystic fluid of benign and malignant ovarian tumors Anticancer Res 2005 25 3A 1581 1589 16033064

[B15] Jeschke U Kuhn C Mylonas I Schulze S Friese K Mayr D Speer R Briese V Richter DU Haase M Development and characterization of monoclonal antibodies for the immunohistochemical detection of glycodelin A in decidual, endometrial and gynaecological tumour tissues Histopathology 2006 48 4 394 406 10.1111/j.1365-2559.2006.02351.x 16487361

[B16] Remmele W Stegner HE [Recommendation for uniform definition of an immunoreactive score (IRS) for immunohistochemical estrogen receptor detection (ER-ICA) in breast cancer tissue] Pathologe 1987 8 3 138 140 3303008

[B17] Lenhard M Lennerova T Ditsch N Kahlert S Friese K Mayr D Jeschke U Opposed roles of follicle-stimulating hormone and luteinizing hormone receptors in ovarian cancer survival Histopathology 2011 58 6 990 994 10.1111/j.1365-2559.2011.03848.x 21585434

[B18] Lenhard M Tsvilina A Schumacher L Kupka M Ditsch N Mayr D Friese K Jeschke U Human chorionic gonadotropin and its relation to grade, stage and patient survival in ovarian cancer BMC Cancer 2012 12 1 2 10.1186/1471-2407-12-2 22214378 PMC3311592

[B19] Hautala LC Greco D Koistinen R Heikkinen T Heikkila P Aittomaki K Blomqvist C Koistinen H Nevanlinna H Glycodelin expression associates with differential tumour phenotype and outcome in sporadic and familial non-BRCA1/2 breast cancer patients Breast Cancer Res Treat 2011 128 1 85 95 10.1007/s10549-010-1065-y 20676758

[B20] Kunert-Keil C Steinmuller F Jeschke U Gredes T Gedrange T Immunolocalization of glycodelin in human adenocarcinoma of the lung, squamous cell carcinoma of the lung and lung metastases of colonic adenocarcinoma Acta Histochem 2011 113 8 798 802 10.1016/j.acthis.2010.11.009 21168900

[B21] Mandelin E Lassus H Seppala M Leminen A Gustafsson JA Cheng G Butzow R Koistinen R Glycodelin in ovarian serous carcinoma: association with differentiation and survival Cancer Res 2003 63 19 6258 6264 14559812

[B22] Dell A Morris HR Easton RL Panico M Patankar M Oehniger S Koistinen R Koistinen H Seppala M Clark GF Structural analysis of the oligosaccharides derived from glycodelin, a human glycoprotein with potent immunosuppressive and contraceptive activities J Biol Chem 1995 270 41 24116 24126 10.1074/jbc.270.41.24116 7592613

[B23] Jeschke U Mylonas I Kunert-Keil C Stahn R Scholz C Janni W Kuhn C Schroder E Mayr D Friese K Immunohistochemistry, glycosylation and immunosuppression of glycodelin in human ovarian cancer Histochem Cell Biol 2009 131 2 283 295 10.1007/s00418-008-0510-z 18853174

[B24] Napoletano C Bellati F Landi R Pauselli S Marchetti C Visconti V Sale P Liberati M Rughetti A Frati L Ovarian cancer cytoreduction induces changes in T cell population subsets reducing immunosuppression J Cell Mol Med 2010 14 12 2748 2759 10.1111/j.1582-4934.2009.00911.x 19780872 PMC3822725

[B25] Zheng W Lu JJ Luo F Zheng Y Feng Y Felix JC Lauchlan SC Pike MC Ovarian epithelial tumor growth promotion by follicle-stimulating hormone and inhibition of the effect by luteinizing hormone Gynecol Oncol 2000 76 1 80 88 10.1006/gyno.1999.5628 10620446

[B26] Minegishi T Kameda T Hirakawa T Abe K Tano M Ibuki Y Expression of gonadotropin and activin receptor messenger ribonucleic acid in human ovarian epithelial neoplasms Clin Cancer Res 2000 6 7 2764 2770 10914722

[B27] Toth B Roth K Kunert-Keil C Scholz C Schulze S Mylonas I Friese K Jeschke U Glycodelin protein and mRNA is downregulated in human first trimester abortion and partially upregulated in mole pregnancy J Histochem Cytochem 2008 56 5 477 485 10.1369/jhc.2008.950600 18256018 PMC2324189

[B28] Jeschke U Karsten U Reimer T Richter DU Bergemann C Briese V Mylonas I Friese K Stimulation of hCG protein and mRNA in first trimester villous cytotrophoblast cells in vitro by glycodelin A J Perinat Med 2005 33 3 212 218 15914343 10.1515/JPM.2005.039

[B29] Dian D Janni W Kuhn C Mayr D Karsten U Mylonas I Friese K Jeschke U Evaluation of a novel anti-mucin 1 (MUC1) antibody (PankoMab) as a potential diagnostic tool in human ductal breast cancer; comparison with two established antibodies Onkologie 2009 32 5 238 244 10.1159/000209280 19420969

[B30] Jaffe RC Donnelly KM Fazleabas AT The induction of baboon glycodelin expression by progesterone is not through Sp1 Mol Hum Reprod 2003 9 1 35 40 10.1093/molehr/gag008 12529418

[B31] Horowitz IR Cho C Song M Flowers LC Santanam N Parthasarathy S Ramachandran S Increased glycodelin levels in gynecological malignancies Int J Gynecol Cancer 2001 11 3 173 179 10.1046/j.1525-1438.2001.01017.x 11437921

[B32] Richter C Baetje M Bischof A Makovitzky J Richter DU Gerber B Briese V Expression of the glycodelin A gene and the detection of its protein in tissues and serum of ovarian carcinoma patients Anticancer Res 2007 27 4A 2023 2025 17649816

[B33] Staebler A Diebold J [Molecular pathology of epithelial ovarian neoplasias: from the phenotype-genotype correlation to new targets in diagnostics and therapy] Pathologe 2007 28 3 180 186 10.1007/s00292-007-0910-1 17431628

[B34] Lang EE Venkatraman G Glycodelin gene expression in human peripheral white blood cells Ir J Med Sci 2007 176 2 101 104 10.1007/s11845-007-0019-9 17570010

[B35] Tulppala M Julkunen M Tiitinen A Stenman UH Seppala M Habitual abortion is accompanied by low serum levels of placental protein 14 in the luteal phase of the fertile cycle Fertil Steril 1995 63 4 792 795 7890064 10.1016/s0015-0282(16)57483-4

[B36] Dalton CF Laird SM Estdale SE Saravelos HG Li TC Endometrial protein PP14 and CA-125 in recurrent miscarriage patients; correlation with pregnancy outcome Hum Reprod 1998 13 11 3197 3202 10.1093/humrep/13.11.3197 9853880

[B37] Loukovaara S Immonen IR Loukovaara MJ Koistinen R Kaaja RJ Glycodelin: a novel serum anti-inflammatory marker in type 1 diabetic retinopathy during pregnancy Acta Ophthalmol Scand 2007 85 1 46 49 17244209 10.1111/j.1600-0420.2006.00766.x

